# Molecular Taxonomy Provides New Insights into *Anopheles* Species of the Neotropical Arribalzagia Series

**DOI:** 10.1371/journal.pone.0119488

**Published:** 2015-03-16

**Authors:** Giovan F. Gómez, Sara A. Bickersmith, Ranulfo González, Jan E. Conn, Margarita M. Correa

**Affiliations:** 1 Grupo de Microbiología Molecular, Escuela de Microbiología, Universidad de Antioquia, Medellín, Antioquia, Colombia; 2 Griffin Laboratory, Wadsworth Center, New York State Department of Health, Singerlands, New York, United States of America; 3 Facultad de Ciencias Naturales y Exactas, Universidad del Valle, Cali, Valle del Cauca, Colombia; 4 Department of Biomedical Sciences, School of Public Health, State University of New York, Albany, New York, United States of America; University of Queensland & CSIRO Biosecurity Flagship, AUSTRALIA

## Abstract

Phylogenetic analysis of partial mitochondrial cytochrome oxidase c subunit I (*COI*) and nuclear internal transcribed spacer 2 (ITS2) sequences were used to evaluate initial identification and to investigate phylogenetic relationships of seven *Anopheles* morphospecies of the Arribalzagia Series from Colombia. Phylogenetic trees recovered highly supported clades for *An*. *punctimacula*s.s., *An*. *calderoni*, *An*. *malefactor* s.l., *An*. *neomaculipalpus*, *An*. *apicimacula* s.l., *An*. *mattogrossensis* and *An*. *peryassui*. This study provides the first molecular confirmation of *An*. *malefactor*from Colombia and discovered conflicting patterns of divergence for the molecular markers among specimens from northeast and northern Colombia suggesting the presence of two previously unrecognized Molecular Operational Taxonomic Units (MOTUs). Furthermore, two highly differentiated *An*. *apicimacula* MOTUs previously found in Panama were detected. Overall, the combined molecular dataset facilitated the detection of known and new Colombian evolutionary lineages, and constitutes the baseline for future research on their bionomics, ecology and potential role as malaria vectors.

## Introduction

Malaria elimination remains a goal in Colombia where 64,309 malaria cases were reported in 2012 [1]. After Brazil, Colombia consistently has the highest number of annual malaria cases in Latin America [2], and underreporting is common [3]. Vector control remains one of the most effective measures to prevent malaria transmission [4,5] and for this, accurate *Anopheles* species identification is an essential part of targeted control strategies [5]. However, in Colombia several species in the subgenus *Anopheles*, including some potential malaria vectors, are relatively understudied.

The *Anopheles* subgenus comprises 187 valid species of which 56 are reported in the New World; 24 of these species are in the Neotropical Arribalzagia Series [6]. All species included in this Series have a unique characteristic wing spot pattern that includes a dark spot at the end of the subcostal vein [7]. Of 47 anopheline species recorded in Colombia [8–10], 14 belong to the *Anopheles* subgenus, of which 12 are in the Arribalzagia Series [11]. Of these, *Anopheles punctimacula* Dyar & Knab and *Anopheles neomaculipalpus* Curry have been considered secondary malaria vectors [12,13]. *Anopheles calderoni* Wilkerson, recently detected in Colombia and Ecuador [14], is a suspected malaria vector [15], based on its anthropophilic behavior in the Colombian Pacific region [16]. *Anopheles mattogrossensis* and *Anopheles peryassui* were both previously found infected with *Plasmodium vivax* and *P*. *falciparum* in Brazil [17], but have not been incriminated in Colombia. A number of these species have been described or re-described based on morphological characters of life stages or male genitalia [18–21], and a few molecular taxonomic studies have been conducted revealing hidden diversity [14,22]. Such information is an essential prerequisite for understanding the biology, bionomics and role in malaria transmission of these species.

Anopheline surveys are mostly based on field collected adult females, using human and animal baits, traps or other methods [23,24]. Rapid and accurate species identification of adult *Anopheles* females is of great relevance for vector biologists, particularly among species presenting difficulties during morphological identification[14,22,25]. Morphological characters of adult females, although useful [8,18,26,27], are limited for discriminating among closely related species or cryptic species with overlapping geographical distributions [28,29]. Morphological similarity among Arribalzagia Series species is widely documented [13,19,20]. For example, a recent molecular study that compared several specimens morphologically defined as *An*. *punctimacula* from Colombia, with reference material from Peru, Ecuador and Panama, revealed that some of these were *An*. *calderoni* [14].

Nuclear and mitochondrial markers have been used in molecular systematic studies and to elucidate phylogenetic relationships among *Anopheles* species [30]. Of these markers, the ITS2 (Internal Transcribed Spacer 2) region is reliable for differentiation of closely related species [31–33] and restriction fragment length polymorphism (RFLP) of the ITS2 is a sensitive, specific and rapid method for molecular confirmation [19,22,25,34–36]. The mitochondrial *COI* barcode region is another important systematics tool, but recent analysis suggests that resolution is higher when the barcode is combined with nuclear markers, at least for mosquitoes [30]. Morphology and *COI* barcode were used to discriminate successfully among *An*. *calderoni*, *An*. *malefactor* and *An*. *punctimacula* in Colombia [14]. Recently, based on phylogenetic analysis of *COI* and ITS2 sequences, *An*. *punctimacula* in Panama was designated as a species complex that includes at least two lineages (*An*. *punctimacula* s.s. and lineage B). Likewise, *An*. *apicimacula* encompasses at least two species, each comprising two lineages [22].

Considering that: i) accurate *Anopheles* species identification is essential for the design of targeted control vector strategies [23], ii) previous molecular work has suggested the presence of species complexes among the Arribalzagia Series, and iii) few studies exist on the molecular taxonomy of these species despite their possible role as malaria vectors, we hypothesize that seven morphospecies represent more than seven Molecular Operational Taxonomic Units (MOTUs) [37] in Colombia.

## Material and Methods

### Specimen sampling and DNA extraction

**Fig 1:**
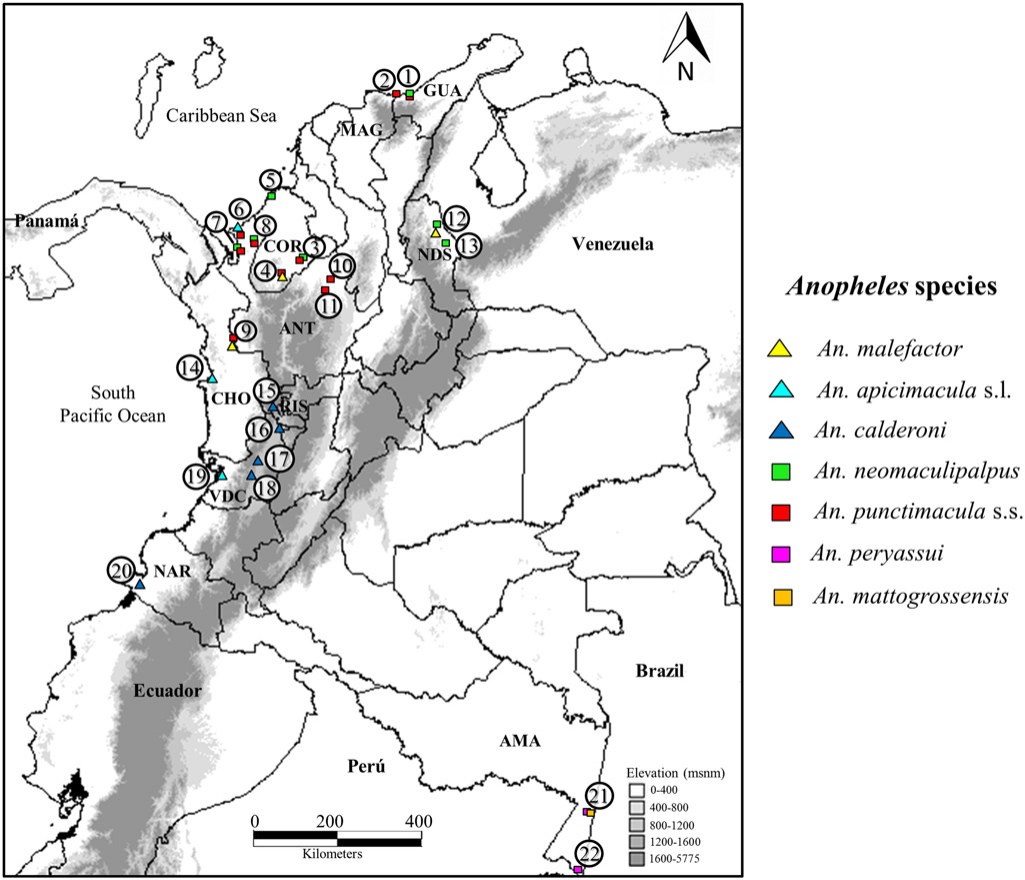
Map of collection sites for Arribalzagia Series species. Departments are noted with three letter codes. AMA: Amazonas; ANT: Antioquia; CHO: Chocó; COR: Córdoba; GUA: La Guajira; MAG: Magdalena; NAR: Nariño; NDS: Norte de Santander; RIS: Risaralda; VDC: Valle del Cauca. Identical symbols indicate the same species. Numbers on the map indicate collections sites detailed in Table 1.

Specimens were collected in various localities across ten departments of Colombia from 2005–2012 and the study did not involve endangered or protected species. Mosquitoes were collected on private property, and permission was received from landowners prior to sampling (Fig. 1, Table 1). Most specimens were collected as adults using human landing catches under a protocol and written informed consent agreement approved by a University of Antioquia Institutional Review Board (Comité de Bioética, Sede de Investigación Universitaria, CBEIH- SIU, approval number 07–41–082). In addition, some *An*. *malefactor* fourth stage larvae were collected and reared to adults. Selected larval exuviae and male genitalia were mounted on microscope slides using Euparal, and at least one voucher specimen per species was deposited in the collection of the Laboratorio de Microbiología Molecular, Universidad de Antioquia, Colombia. Morphospecies were identified using the keys of González & Carrejo [8] and Wilkerson & Strickman [26]. Genomic DNA was extracted from abdomens using a salt precipitation protocol [38].

**Table 1:** Detailed information on collected specimens of Arribalzagia Series species from Colombian localities.

Species	*n*	Department	Municipality	Map No.^a^	Sampling sites	Collection method(s)^b^	Collection Year(s)	Latitude	Longitude	ITS2	*COI*
*An*. *apicimacula* s.l.	4	Antioquia	Necoclí	6	Pueblo Nuevo-Bellavista	HLC	2009	8.426	-76.784	4	4
	5	Chocó	Nuquí	14	Panguí	HLC	2006	5.700	-77.267	2	3
	3	Valle del Cauca	Buenaventura	19	Zacarias-La Balastrera	HLC	2009	3.817	-76.983	1	3
*An*. *calderoni*	23	Nariño	Tumaco	20	Pindale	HLC, RC	2009–2010	1.617	-78.733	5	19
	5	Risaralda	Pereira	15	La Carbonera	HLC	2012	4.878	-75.858	3	4
	17	Valle del Cauca	Buga	18	Laguna de Sonso	HLC	2012	3.876	-76.348	3	16
	12	Valle del Cauca	Riofrio	17	El Jagual	HLC	2012	4.143	-76.279	3	12
	4	Valle del Cauca	Cartago	16	Hacienda Limones	CBC	2012	4.943	-75.903	2	4
*An*. *malefactor*	4	Antioquia	Vigía del Fuerte	9	San Antonio de Padua	BS	2010	6.283	-76.750	1	4
	1	Córdoba	Puerto Libertador	4	Corregimiento Juan José	HLC	2010	7.717	-75.850	0	1
	1	Norte de Santander	Tibú	12	Caño Victoria	HLC	2012	8.569	-72.667	1	1
*An*. *neomaculipalpus*	9	Antioquia	San Pedro de Urabá	8	El Caño	HLC, CBC	2011–2012	8.283	-76.383	3	9
	1	Antioquia	Turbo	7	Yarumal	HLC	2008	8.117	-76.733	1	1
	14	Córdoba	Moñitos	5	Rio Cedro	HLC	2005–2006	9.250	-76.100	5	14
	2	Córdoba	Montelíbano	3	Montelibano-Rural	HLC	2012	7.983	-75.417	0	2
	1	La Guajira	Dibulla	1	Dibulla-Rural	HLC	2011	11.267	-73.300	0	1
	2	Norte de Santander	Zulia	13	Santa Rosa	CBC	2011	8.250	-72.550	2	2
	1	Norte de Santander	Tibú	12	Caño Victoria	HLC	2012	8.569	-72.667	1	1
*An*. *punctimacula*	5	Antioquia	San Pedro de Urabá	8	El Caño	HLC, CBC	2010–2012	8.283	-76.383	1	4
	2	Antioquia	Necoclí	6	Pueblo Nuevo-Bellavista	HLC	2009	8.426	-76.784	1	2
	2	Antioquia	El Bagre	10	La Capilla	HLC	2009	7.583	-74.817	1	1
	1	Antioquia	Turbo	7	Camerún	HLC	2007	8.133	-76.717	1	0
	1	Antioquia	Vigía del Fuerte	9	San Antonio de Padua	HLC	2010	6.283	-76.750	1	0
	1	Antioquia	Zaragoza	11	El Retiro	HLC	2008	7.483	-74.850	1	0
	3	Córdoba	Montelíbano	3	Montelibano-Rural	HLC	2012	7.983	-75.417	0	3
	1	Córdoba	Puerto Libertador	4	Corregimiento Juan José	CBC	2010	7.717	-75.850	0	1
	4	La Guajira	Dibulla	1	Dibulla-Rural	HLC	2011	11.267	-73.300	4	4
	2	Magdalena	Los Achiotes	2	Los Achiotes	HLC	2005	11.250	-73.600	2	0
*An*. *mattogrossensis*	2	Amazonas	Tarapacá	21	Nueva Unión	HLC	2010	-2.896	-69.758	2	2
*An*. *peryassui*	3	Amazonas	Leticia	22	Km 6 and Km 11	HLC	2009	-4.215	-69.933	1	3
	2	Amazonas	Tarapacá	21	Nueva Unión	HLC	2010	-2.896	-69.758	2	0
**Total**	**138**									**54**	**121**

^a^ Map number refers to the municipality as depicted in Fig. 1.

^b^ HLC: Human-Landing Collection; CBC: Cattle-Bait Collection; BS: Breeding Site- larvae reared to adult; RC: Resting Collection.

### Barcode region

The *COI* gene region was amplified using the LCO and HCO universal primers [39] and modified PCR conditions [40]. PCR products were subjected to bidirectional sequencing. All the sequences were translated to amino acids to detect stop codons and potential shifts in reading frame as a test for possible nuclear mitochondrial pseudogenes (Numts). The *COI* protein sequence published for *Anopheles gambiae* (GI: 5834913) was used as a reference to indicate positions of amino acid changes. Potential contamination was explored using BLAST searches [41] (http://blast.ncbi.nlm.nih.gov/Blast.cgi). Additional *COI* sequences from GenBank were included for comparison with the original dataset (Table 2). *COI* sequences obtained in this work were deposited in GenBank under accession numbers KF698801- KF698878.

**Table 2:** Species and GenBank accession numbers of *COI* and ITS2 sequences used in the phylogenetic analyses.

*Anopheles* species	*COI*	ITS2
*An*. *punctimacula*	HQ622626, KC354818, KF698833-KF698837	JX212806-JX212814, KF698889-KF698895
*An*. *malefactor*	HQ622625, KF698838-KF698842	JX212822-JX212823, KF698896-KF698897
*An*. *neomaculipalpus*	JX205124, JX205125, KF698843-KF698864	JX212821, KF698898-KF698906
*An*. *apicimacula*	KF698866-KF698872	JX212815-JX212819, KF698907-KF698910, KM262754-KM262760
*An*. *mattogrossensis*	JX205126, KF698873-KF698874	KF698917-KF698920
*An*. *peryassui*	HM022405, KF698875-KF698877	KF698911-KF698916
*An*. *calderoni*	HQ642964-HQ642974, KF698801-KF698832,	KF698879-KF698888
*An*. *pseudopunctipennis*	KF698878	KF698921

Sequences obtained in this work are underlined.

### ITS2 region

The rDNA ITS2 was amplified using the primers ITS2-F (5’-TGAACTGCAGGACACATGAAC-3’) and ITS2-R (5’-ATGCTTAAATTTAGGGGGTAGTC-3’), and analyzed by a RFLP assay [25,34,36]. Available confirmed specimens from the Arribalzagia Series species were used as controls for amplification and RFLP. The *in silico* restriction digestion for each species was predicted using Webcutter 2.0 tool available at http://rna.lundberg.gu.se/cutter2.

PCR products were cloned using the CloneJET PCR Cloning Kit (Thermo Fisher Scientific Inc., PA, USA), and three to five clones per specimen were selected for sequencing. The ITS2 sequences were deposited in GenBank under accession numbers KF698879- KF698921 and KM262754-KM262760.

### *COI* and ITS2 analyses

The *COI* and ITS2 sequences were edited in Geneious version 6.0.6 [42]. For ITS2, the flanking 5.8S and 28S regions were identified using the Diptera model through the ITS2 annotation tool [43,44] and excluded before ITS2 analysis. Analyses of intragenomic, intra- and interspecific ITS2 variation were performed in at least two specimens per morphospecies using DAMBE [45]. Mean uncorrected pairwise distances and standard errors were calculated with MEGA 5.0 [46]. The presence of interspersed and tandem repeat sequences was explored using the bioinformatics software Spectral Repeat Finder (SRF) [47] and Tandem Repeat Occurrence Locator (TROLL) [48], respectively.

For interspecific comparison, ITS2 sequences of *Anopheles* species belonging to Arribalzagia Series retrieved from GenBank also were analyzed (Table 2). Manual editing and multiple sequence alignment were performed with Geneious version 6.0.6 under default parameters. Sequences were checked for insertions or deletions.

### Phylogenetic analysis

Sequences were aligned and gaps were treated as missing data. Because saturation in substitutions can have negative effects on phylogenetic inference, saturation levels were tested in DAMBE [49]. The best-fit model of DNA substitution and the parameter estimates used for tree construction were chosen according to the Akaike Information Criterion (AIC) as implemented in jModeltest version 2.1.4 [50]. The results provided TrN+I+G and TIM3+G as the best-fit models for the *COI* and ITS2 datasets, respectively. Phylogenetic trees based on ITS2, *COI* or the concatenated (*COI*+ITS2) datasets were constructed using methods of Neighbor-Joining (NJ) in MEGA 5.0 [46], Maximum Likelihood (ML) in PhyML 3.0 [51] and Bayesian (BI) analysis in MrBayes [52]. To construct the NJ tree we chose Kimura’s (1980) two-parameter model (K2P), typically used in the DNA barcode strategy [53]. ML and BI were run with the parameters inferred from jModeltest.

Although all ITS2 variants were included in the ITS2 phylogeny, only the most frequent variant of each specimen was included in the concatenated analysis. For Bayesian inference, the analyses were initiated with random starting trees and the Markov chain Monte Carlo search was run with four chains for five million (ITS2 and ITS2+*COI*) for ten million generations (*COI*), sampling every 1,000 generations and discarding and average of 25% of each run as burn-in. Bootstrap sampling (1,000 replicates) was performed to test inferred phylogenies. *Anopheles* (*Anopheles*) *pseudopunctipennis* was used as the outgroup [54].

### DNA-based approaches for species recognition

Molecular Operational Taxonomic Units (MOTUs) were identified according to the reciprocal monophyly criterion for the different phylogenetic approaches using individual markers (*COI* or ITS2) or concatenated (*COI*+ITS2) trees. The species delimitation plugin for Geneious [55] was used to calculate Rosenberg’s *P*_*AB*_ value, a test for taxonomic distinctiveness based on the null hypothesis that the observed monophyly was found by chance alone [56]. Correspondence between morphospecies and MOTUs in the gene trees was evaluated.

Criteria for assessing and comparing the *COI* barcode for specimens of the seven Arribalzagia Series species, included Best Match (BM), Best Close Match (BCM) and All Species Barcodes (ASB), as performed in TaxonDNA. These three algorithms are used to test identification success [57]. The presence or absence of the “barcode gap”, or the result of a smaller intraspecific divergence with respect to interspecific divergence, was evaluated. In addition, identification success was determined based on the 1% standard threshold cut-off value suggested by The Barcode of Life Data System [58], using SPIDER [59]. Automatic Barcode Gap Discovery (ABGD) allowed the partitioning of the DNA sequence datasets into clusters of like taxa setting a range of maximum values of intraspecific divergence (*P*) without an *a priori* species hypothesis [60].

## Results

### Species assignment

Overall, molecular confirmation agreed with the morphological identification for 83.21% of the wild-caught females. For the remaining 16.79%, missassignments among individual species were 90.91% (*n* = 10) for *An*. *apicimacula* s.l., 30% (*n* = 9) for *An*. *neomaculipalpus*, 16.67% (*n* = 1) for *An*. *malefactor*, 9.09% (*n* = 2) for *An*. *punctimacula* and 1.64% (*n* = 1) for *An*. *calderoni*. *Anopheles apicimacula* s.l. was frequently confused with *An*. *punctimacula* (54.55%) or *An*. *neomaculipalpus* (36.63%). The latter species was also misidentified as *An*. *punctimacula* (23.33%).

The PCR-RFLP-ITS2 assay was used as an initial approach for species confirmation. The PCR-ITS2 yielded a different product size for each species (Table 2), ranging from 393 bp for *An*. *punctimacula* to 481 bp for *An*. *apicimacula* s.l. Interestingly, there were two slightly different PCR product sizes for *Anopheles malefactor* Dyar and Knab: 396 bp (specimens from Antioquia) and 401 bp (specimen from Norte de Santander). The species *An*. *punctimacula* s.s., *An*. *apicimacula*, *An*. *neomaculipalpus* could be differentiated by their PCR-RFLP-ITS2 patterns (Table 3). Furthermore, *An*. *mattogrossensis* and *An*. *peryassui* yielded similar banding patterns with slight differences in band sizes, which could not be discriminated on the electrophoresis gel. Two restriction patterns were detected for *An*. *malefactor*. Specimens from Antioquia and Córdoba Departments yielded a similar restriction pattern to *An*. *punctimacula* s.s. However, the ITS2 fragment corresponding to the Norte de Santander specimen was not cut by the enzyme, a result confirmed by bioinformatic analysis.

**Table 3:** Comparison of *in vitro* with *in silico* results from PCR-RFLP-ITS2 assay.

Species	Agarose gel fragment sizes (bp)		Bioinformatic prediction of fragment sizes (bp)	
	PCR product	PCR-RFLP	PCR product	PCR-RFLP
*An*. *neomaculipalpus*	500	259, 165, 111	452	255, 136, 61
*An*. *apicimacula* lineage C (Caribbean)	481	300, 150, 70	481	290, 130, 61
*An*. *apicimacula* lineage P (Pacific)	480	390, 70	480	372, 61, 47
*An*. *punctimacula* s.s.	397	334, 81	393	317, 76
*An*. *calderoni*	394	Uncut	401	Uncut
*An*. *malefactor*	399	Uncut; 378, 313, 113	401; 396	Uncut; 326, 70
*An*. *mattogrossensis*	505	230, 80	464*	213, 190, 61
*An*. *peryassui*	500	300, 96	464*	287, 116, 61

* An ITS2 variant of 463 bp was found that yielded 286, 116 and 61 bp in the *in silico* restriction analysis.

PCR: Polymerase Chain Reaction; RFLP: Restriction Fragment Length Polymorphisms; (bp): base pairs.

### ITS2 sequence characterization

The Arribalzagia Series species had a range of ITS2 size from 265 bp in *An*. *punctimacula* s.s. to 353 bp in *An*. *apicimacula* from the Colombian Caribbean or lineage C. Overall, ITS2 size was conserved at the intraspecific level, except for a few specimens within some species (S1 Table). At the individual level, low intragenomic ITS2 variation was detected in all species and was restricted to a few mutations. The highest ITS2 intragenomic mean uncorrected *p*-distance of 1.23% was detected for one *An*. *calderoni* specimen from Risaralda. *Anopheles mattogrossensis* specimens had two ITS2 variants that differed by one bp (336 and 337 bp); the mean uncorrected *p*-distance between them was 1.19%. Comparison of both ITS2 variants with those available from GenBank found that the 336 bp ITS2 variant had the same deletion as a sequence reported for *An*. *mattogrossensis* from the state of Rondônia in western Brazil (AF461754). Likewise, the 337 bp variant was similar to the one reported for *An*. *mattogrossensis* from southern Colombia (JX198307). Further details about the specimens, number of clones and the mean uncorrected *p*-distance among ITS2 sequences can be found in S1 Table.

At the intraspecific level, two ITS2 variants were detected in *An*. *malefactor*. The ITS2 variant detected in Norte de Santander Department, NE Colombia, was identical to a previously reported ITS2 for a Panamanian specimen of *An*. *malefactor*, 273 bp in length (JX212823). In contrast, specimens from a Caribbean locality in Antioquia Department, NW Colombia, had a deletion of five base pairs (268 bp) and 12 single mutations. The uncorrected *p-*distance between ITS2 variants of *An*. *malefactor* from the NE and NW was 4.5%. The overall mean uncorrected *p*-distance among all ITS2 sequences for each MOTU was: *An*. *neomaculipalpus* (0.2±0.1%), *An*. *punctimacula* s.s. (0.3±0.2%), *An*. *calderoni* (0.3±0.1%), *An*. *peryassui* (0.5±0.2%), *An*. *mattogrossensis* (0.8±0.4%), *An*. *apicimacula* s.l. (0.9±0.2%) and *An*. *malefactor* (2.4±0.6%). *Anopheles punctimacula* s.s. was molecularly confirmed in Caribbean and northwest Colombian localities by comparison of ITS2 sequences with those from Panama. Three fixed substitutions detected in the Panamanian specimens (positions 62, 131 and 81 of the ITS2 alignment) [22], were also found in those from Colombia. Two additional nucleotide substitutions were detected, one transversion at position 108 of the ITS2 alignment (T→A) in some sequences from Antioquia and La Guajira, and a unique sequence with a transition (G→A) at position 169 from La Guajira. The mean average ITS2 interspecific K2P distance was 48.8%. The highest ITS2 genetic distance was between *An*. *peryassui* and *An*. *mattogrossensis* (66.2±3%), and the lowest between *An*. *punctimacula* s.s. and *An*. *malefactor* (11.5±1.8%) (S2 Table). A common pentanucleotide tandem repeat (CACCT)_2_ present in all ITS2 of the Arribalzagia Series species from Panama [22], was detected in five Colombian species, *An*. *punctimacula* s.s., *An*. *calderoni*, *An*. *malefactor*, *An*. *neomaculipalpus* and *An*. *apicimacula* s.l. Additionally, one hexanucleotide tandem repeat (TGCGCA)_2_ was detected in *An*. *calderoni*.

### DNA barcoding

The *COI* alignment was 611 bp and yielded 79 unique haplotypes. There were 180 polymorphic sites (29.46%), from which 160 were parsimoniously informative (26.18%). Nucleotide changes mainly occurred at the third-codon positions and were silent. However, some interspecific *COI* nucleotide differences led to non-synonymous amino acid substitutions and some single amino acid differences were fixed at the species level. *Anopheles punctimacula* s.s. had a substitution (serine to alanine) at position 171, and *An*. *malefactor* at position 191 (valine to isoleucine). The average genetic distance among all *COI* sequences was 9.77±0.83%. Intraspecific variation values were *An*. *punctimacula* s.s. (0.42±0.16%), *An*. *malefactor* (0.55±0.2%), *An*. *peryassui* (0.83±0.26%), *An*. *neomaculipalpus* (0.86±0.19%), *An*. *mattogrossensis* (1±0.33%), *An*. *calderoni* (1.09±0.21%) and *An*. *apicimacula* s.l. (4.44±0.63%).

The most frequent *An*. *calderoni* haplotype (KF698801 = 27.27%) was shared among Pacific region localities within 122 km (Buga-Pereira). For *An*. *punctimacula* s.s., the most frequent haplotype (KF698833 = 66.67%) was from localities in Antioquia, Córdoba and La Guajira Departments (maximum straight-line distance between the farthest localities = 495 km). Lastly, the localities of Antioquia and Córdoba contained the most frequent haplotype for *An*. *neomaculipalpus* (KF698843 = 20%). There was a high number of unique haplotypes in the analyzed taxa (49.6%). The overall mean nucleotide diversity for the barcode was 0.084.

**Fig 2:**
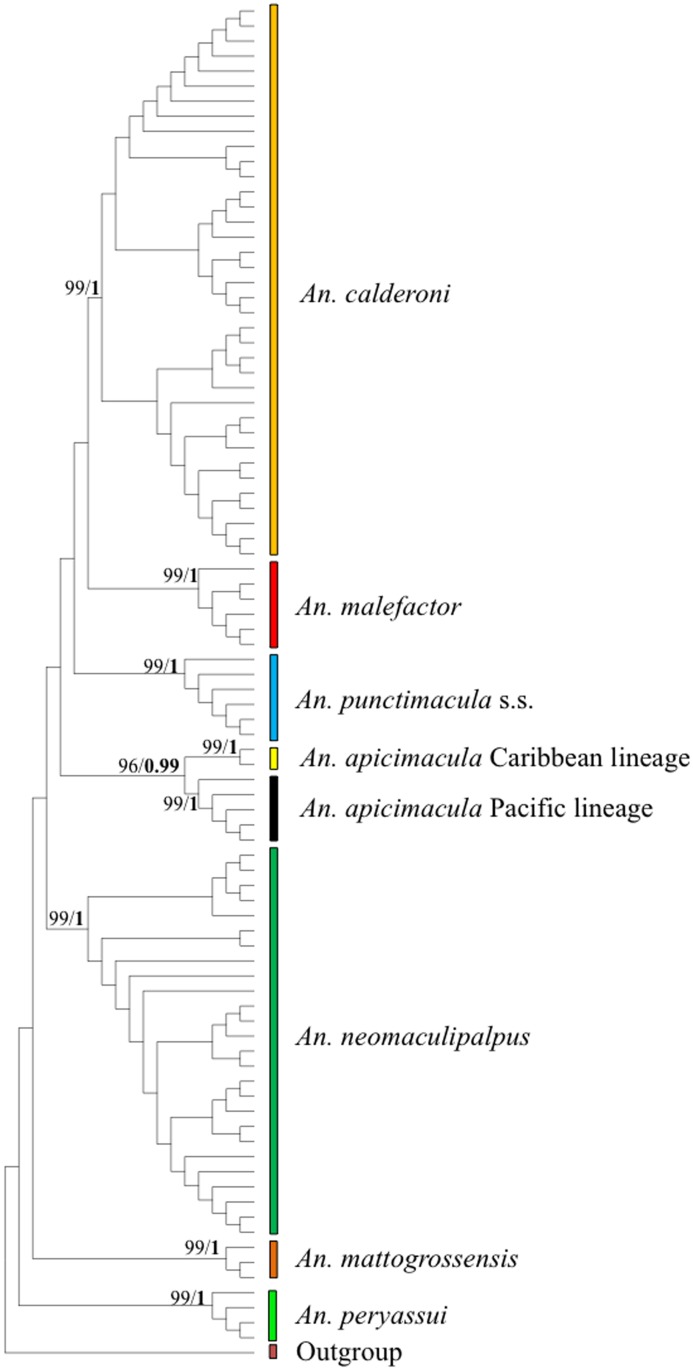
NJ topology based on barcode (*COI*) sequences for members of the Arribalzagia Series. First number in each node indicate NJ bootstrap values (in percentages), numbers in bold indicate Bayesian posterior probability for each MOTU. *Anopheles pseudopunctipennis* was included as the outgroup.

Intertaxa *COI* genetic distances among the seven Arribalzagia Series members ranged from 9.3±1.3% between *An*. *punctimacula* s.s. and *An*. *malefactor*, to 14.7±0.8% between *An*. *calderoni* and *An*. *peryassui* (S3 Table). Each of the *Anopheles* members formed a monophyletic group in the NJ, ML and BI trees with high support (Fig. 2). Results were also supported by Rosenberg’s *P*_*AB*_ values that were significant for all MOTUs (*p*<0.05). The BM and BCM criteria yielded identical results with 100% “correct” identifications. Moreover, 97.87% and 2.13% of the *COI* sequences were assigned as “correct” and “ambiguous” using the ASB criterion, respectively. The problematic identifications corresponded only to *COI* sequences of *An*. *apicimacula* lineage C.

A *COI* threshold value equal or greater than 1% provided a perfect species identification for the dataset, with the presence of a barcoding gap. The ABGD method consistently revealed eight groups using an *a priori* intraspecific genetic divergence ≤1.47% under Jukes and Cantor’s model (JC69), supporting each morphospecies as a single species, except for *An*. *apicimacula* that encompassed two provisional MOTUs for all lower cut-offs.

### Phylogenetic relationships

**Fig 3:**
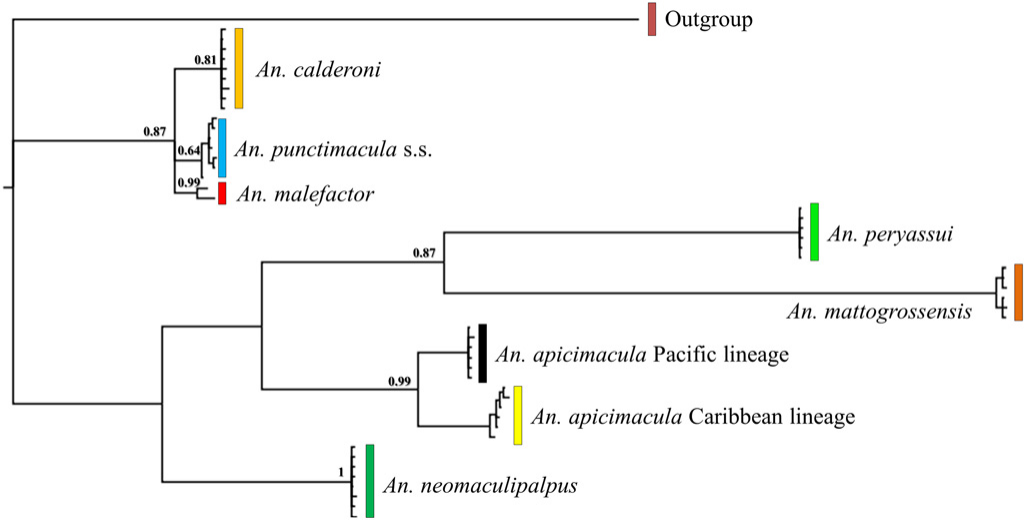
Bayesian topology of ITS2 sequences for species of the Arribalzagia Series. Numbers on each branch represent posterior probabilities. *Anopheles pseudopunctipennis* was the outgroup.

**Fig 4:**
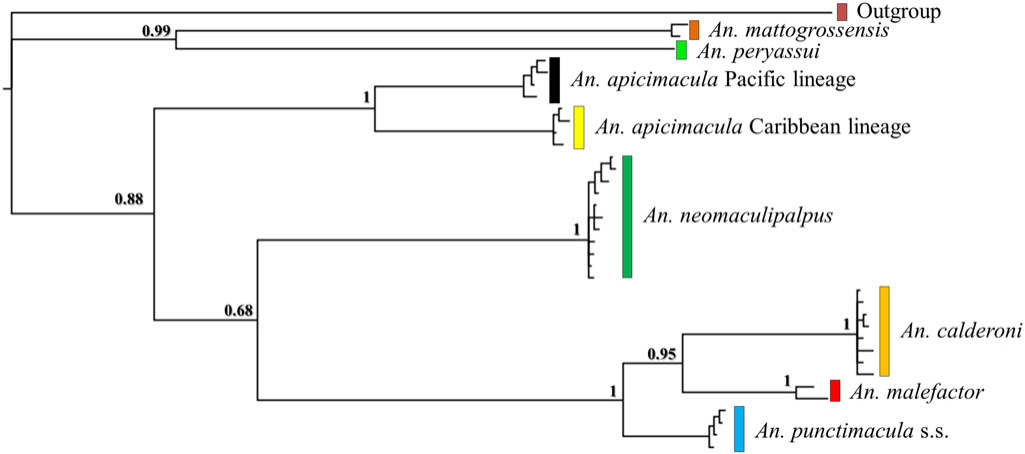
Bayesian topology of the concatenated *COI* and ITS2 datasets. Numbers on each branch represent posterior probabilities. *Anopheles pseudopunctipennis* was the outgroup.

There was no saturation signal among the *COI* sequences (*p* < 0.05), validating the dataset for phylogenetic analyses. Phylogenetic trees based on NJ, ML and BI approaches with each marker (i.e. *COI* or ITS2) showed highly supported discrete clades for *An*. *punctimacula* s.s., *An*. *calderoni*, *An*. *malefactor*, *An*. *neomaculipalpus*, *An*. *apicimacula* Caribbean and Pacific lineages, *An*. *mattogrossensis* and *An*. *peryassui*. Two highly supported mitochondrial lineages (BPP: 0.98) were detected in all trees for *An*. *apicimacula* s.l. that corresponded exclusively to specimens from the Caribbean and Pacific regions of Colombia (Figs. 2–4). Bayesian trees derived from ITS2 and *COI*+ITS2 data showed very similar topologies, whereas species groups in the *COI* tree were less evident.

## Discussion

This study confirms that previously recognized morphospecies of the Arribalzagia Series of Colombia constitute independent evolutionary lineages or MOTUs and reveals hidden lineages. Strong molecular evidence supports at least two geographically separated MOTUs of *An*. *apicimacula* in the Colombian Pacific and Caribbean regions, respectively. The level of *COI* intraspecific variation for *An*. *apicimacula* s.l. (4.44%), compared to the standard value usually found for *Anopheles* species (<2%) [61,62], and the fixed ITS2 sequence differences together support the hypothesis that *An*. *apicimacula* is a complex [22]. These lineages are distributed along the Chocó/Darien/Western Ecuador biodiversity hotspot [63]; a variety of factors in this region, including landscape heterogeneity, historical demographical processes and Pleistocene environmental changes might have driven divergence [64,65]. It will be interesting to include *An*. *apicimacula* specimens from the type locality (Livingston, Guatemala) [66], to determine whether either of these lineages constitutes *An*. *apicimacula* s.s. Further sampling of *An*. *apicimacula* s.l., and the application of integrative taxonomic analysis will assist new species delimitation and geographical range [67,68].

Low intragenomic ITS2 variation was detected for most of the Arribalzagia species (<1%), with values comparable to those for other *Anopheles* species such as African *Anopheles arabiensis* (0.07%), *An*. *gambiae* (0.43%) [69], Neotropical *An*. *nuneztovari* (<0.2%) [70], and members of the Albitarsis Complex (<0.57%) [71]. At the intraspecific level, an unexpected 4.5% divergence between the N and the NE *An*. *malefactor* ITS2 variants was higher than those reported among 21 species of the subgenus *Nyssorhynchus* (0–2.8%) [72] or members of the Albitarsis Complex (0.28–1.17%) [71]. Nonetheless, ITS2 divergence was not supported by *COI* analysis (K2P distance ˂1%). Although the greatest distance between the collection localities for the Colombian *An*. *malefactor* specimens was 517 km, geographical distance alone cannot explain the ITS2 differentiation. The Panamanian *An*. *malefactor* ITS2 sequences were identical to those of the Colombian NE, collected 550 km away, whereas the NW specimens collected 227 km from the Panamanian *An*. *malefactor* collection site differed. Other factors may be responsible for this divergence, e.g., although it is recognized that concerted evolution homogenizes the ITS2 at the species level [73,74], differences in population size or migration rates could also affect ITS2 evolution among populations [75]. Similar *COI* but highly divergent ITS2 sequences in *An*. *malefactor* is hypothesized to be the result of mitochondrial introgression or incomplete lineage sorting at the mitochondrial locus, as observed for other species such as *An*. *cruzi* [76–79] and it also suggests that *An*. *malefactor* comprises at least two MOTUs. An integrative taxonomic approach that includes analysis of additional molecular markers should provide details of population structure, demographic history, and the formation of evolutionarily independent lineages in *An*. *malefactor* [64,80].

Morphological keys to adult females were useful for initial species identification, but some *An*. *apicimacula* were misidentified as *An*. *punctimacula* or *An*. *neomaculipalpus*. Overall, some Arribalzagia Series species females differ in morphological characters such as wing spot pattern (S1 Fig.). For instance, the small and appressed dark scales on the cubital vein of *An*. *apicimacula* s.l., which differ in form in *An*. *punctimacula* and *An*. *neomaculipalpus*, [8,26] was not easily detected in some specimens, resulting in their misidentification. This character of *An*. *apicimacula* is only shared with *An*. *intermedius*, a species that has been historically reported in forested areas from Central and South America [81], but with just one doubtful early record in Villavicencio, Colombia [8,82]. In this study, *An*. *malefactor* s.l. was relatively easy to separate based on its entirely white hindtarsomere 5, which usually has at least one dark band in *An*. *punctimacula* and *An*. *calderoni* [8]. Misidentification of specimens could result from intraspecific variation in wing spots as documented for other anopheline species [14,83], the loss of thoracic scales due to the sampling technique or during the mosquito life cycle [84] or human error in identifying ambiguous or damaged field samples. It will be important to determine if *An*. *malefactor* and *An*. *apicimacula* lineages are truly morphologically cryptic [85].

The PCR-RFLP-ITS2 strategy facilitated the identification of most members of the Arribalzagia Series. However, for accurate identification of *An*. *punctimacula* s.s.-*An*. *malefactor* s.l. and *An*. *mattogrossensis-An*. *peryassui*, that showed similar restriction patterns, we recommend sequencing the ITS2 or *COI* barcode region. Nevertheless, the low cost and effort needed to implement PCR-RFLP protocols in the laboratory [25,34,86,87], suggest the importance of designing a PCR-RFLP strategy based on a useful marker for the rapid and accurate discrimination of species and lineages in the Arribalzagia Series.

## Conclusions

Nuclear and mitochondrial markers recovered monophyletic morphospecies in the Arribalzagia Series and allowed updating records of these species in several localities of the country. This is the first work in Colombia providing molecular confirmation of *An*. *apicimacula*, *An*. *punctimacula* s.s. and *An*. *malefactor* s.l. The two *An*. *apicimacula* evolutionary lineages detected, Pacific and Caribbean, with fixed differences in the mitochondrial and nuclear loci, likely represent two species. A possible mitochondrial introgression event or incomplete lineage sorting during the phylogenetic history of *An*. *malefactor* is hypothesized. Information on accurate identification of Colombian Arribalzagia Series species constitutes the baseline for future studies on their bionomics and vectorial importance that could be used for targeted and effective control efforts.

## Supporting Information

S1 Fig Comparison of wing-spot patterns among five species in the Arribalzagia Series. PSD: presectorial dark; SD: sector dark; PD: preapical dark; AD: apical dark; CuA vein: Cubital vein.
(TIF)

S1 Table Intragenomic variability of ITS2 in analyzed Arribalzagia Series species. (DOCX)

S2 Table ITS2-Interspecific K2P genetic distances. ITS2: Internal transcribed spacer 2. D: genetic distance. SE: Standard Error. K2P: Kimura 2 parameter, used as the evolutionary model.
(DOCX)

S3 Table *COI*-Interspecific K2P genetic distances. *COI*: cytochrome oxidase I gene. D: genetic distance. SE: Standard Error. K2P: Kimura 2 parameter, used as the evolutionary model.
(DOCX)
