# Supplementary figures and images for: Molecular Taxonomy Provides New Insights into Anopheles Species of the Neotropical Arribalzagia Series
Source: PLoS One. 2015 Mar 16;10(3):e0119488. doi: 10.1371/journal.pone.0119488 (PMC4361172; doi:10.1371/journal.pone.0119488)

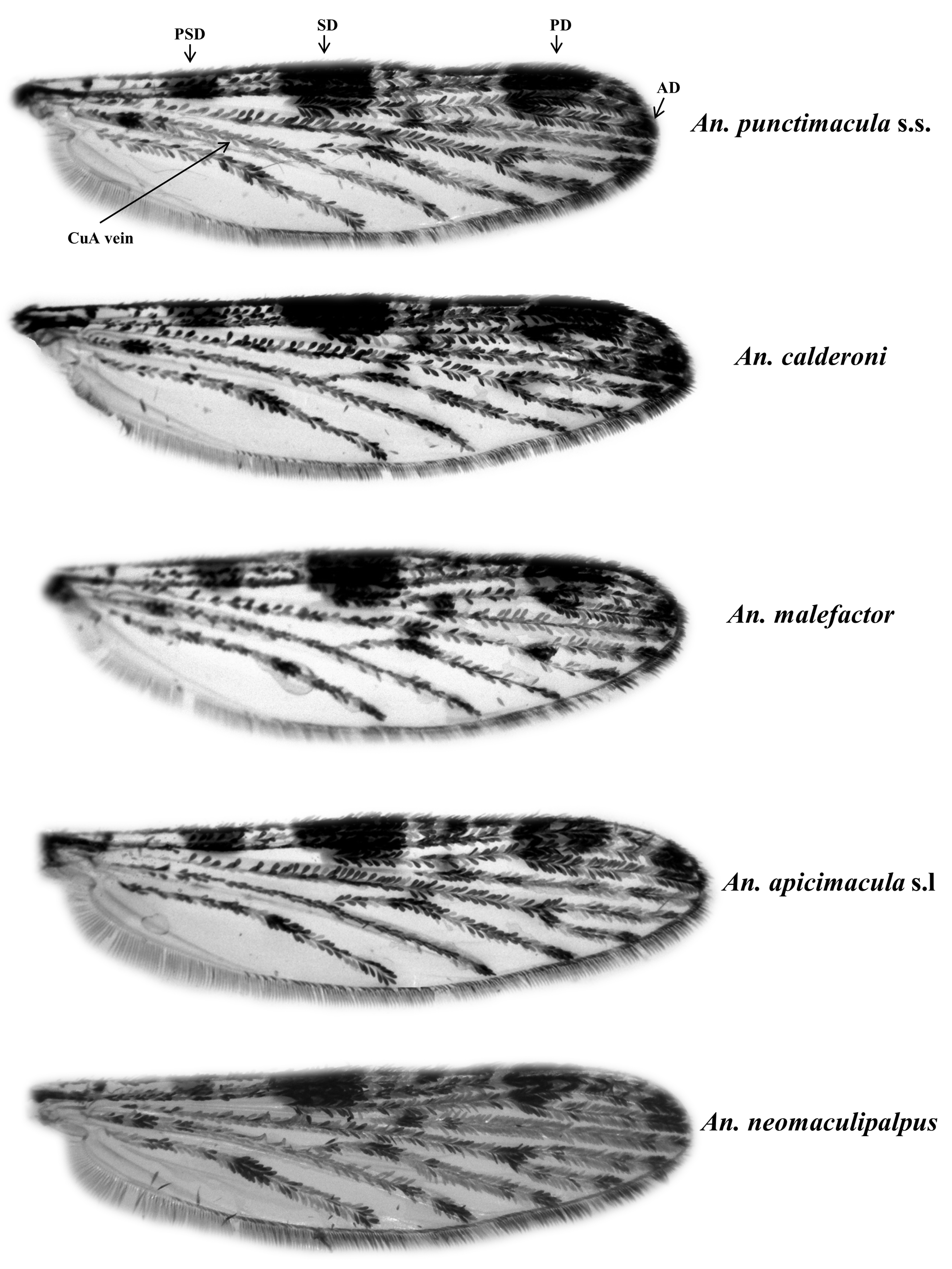

Supplement: S1 Fig — PSD: presectorial dark; SD: sector dark; PD: preapical dark; AD: apical dark; CuA vein: Cubital vein. (TIF) [file pone.0119488.s001.tif]
