# Supplementary material for: Molecular Taxonomy Provides New Insights into Anopheles Species of the Neotropical Arribalzagia Series
Source: PLoS One. 2015 Mar 16;10(3):e0119488. doi: 10.1371/journal.pone.0119488 (PMC4361172; doi:10.1371/journal.pone.0119488)
Supplement: S1 Table — (DOCX) [file pone.0119488.s002.docx]

**Table S1. Intragenomic variability of ITS2 in analyzed Arribalzagia Series species.**

| **Specimen code** | **Morphospecies** | **ITS2**  **length (bp)** | **Clones (n)** | **ITS2 variants (n)** | **Mean uncorrected**  ***p*-distance** | **SE** |
| --- | --- | --- | --- | --- | --- | --- |
| ACH-06 | *An. punctimacula* | 265 | 5 | 2 | 0.0025 | 0.0024 |
| ACH-08 | *An. punctimacula* | 265 | 3 | 1 | 0.0000 | 0.0000 |
| BAG-148 | *An. punctimacula* | 265 | 3 | 3 | 0.0050 | 0.0033 |
| TUR-80 | *An. punctimacula* | 265 | 4 | 2 | 0.0019 | 0.0018 |
| DIB-140 | *An. punctimacula* | 265 | 5 | 2 | 0.0023 | 0.0022 |
| DIB-141 | *An. punctimacula* | 265 | 4 | 4 | 0.0082 | 0.0040 |
| DIB-76 | *An. punctimacula* | 265 | 2 | 1 | 0.0000 | 0.0000 |
| DIB-8 | *An. punctimacula* | 265 | 4 | 2 | 0.0025 | 0.0025 |
| NEC-18 | *An. punctimacula* | 265 | 3 | 2 | 0.0025 | 0.0024 |
| VIF-1655 | *An. punctimacula* | 265 | 3 | 2 | 0.0025 | 0.0025 |
| ZAR-84 | *An. punctimacula* | 265 | 3 | 2 | 0.0025 | 0.0025 |
| SPU-1368 | *An. punctimacula* | 265 | 1 | 1 | 0.0000 | 0.0000 |
| BUG-13 | *An. calderoni* | 273 | 3 | 1 | 0.0000 | 0.0000 |
| TUM-1087 | *An. calderoni* | 273 | 4 | 1 | 0.0000 | 0.0000 |
| TUM-1815 | *An. calderoni* | 273 | 2 | 1 | 0.0000 | 0.0000 |
| TUM-1965 | *An. calderoni* | 272 | 4 | 1 | 0.0000 | 0.0000 |
| TUM-1973 | *An. calderoni* | 273 | 3 | 1 | 0.0000 | 0.0000 |
| TUM-2305 | *An. calderoni* | 273 | 2 | 1 | 0.0000 | 0.0000 |
| BUG-467 | *An. calderoni* | 273 | 3 | 2 | 0.0025 | 0.0023 |
| BUG-476 | *An. calderoni* | 273 | 4 | 2 | 0.0018 | 0.0018 |
| RIO-5 | *An. calderoni* | 273 | 3 | 3 | 0.0049 | 0.0034 |
| RIO-8 | *An. calderoni* | 273 | 3 | 3 | 0.0049 | 0.0034 |
| RIO-21 | *An. calderoni* | 273 | 1 | 1 | 0.0000 | 0.0000 |
| **Specimen code** | **Morphospecies** | **ITS2**  **length (bp)** | **Clones (n)** | **ITS2 variants (n)** | **Mean uncorrected**  ***p*-distance** | **SE** |
| PER-24 | *An. calderoni* | 273 | 3 | 3 | 0.0123 | 0.0123 |
| PER-36 | *An. calderoni* | 273 | 2 | 2 | 0.0037 | 0.0036 |
| PER-66 | *An. calderoni* | 273 | 2 | 1 | 0.0000 | 0.0000 |
| CAR-7 | *An. calderoni* | 273 | 3 | 2 | 0.0025 | 0.0024 |
| CAR-8 | *An. calderoni* | 273 | 3 | 1 | 0.0000 | 0.0000 |
| VIF-472 | *An. malefactor* | 268 | 4 | 1 | 0.0000 | 0.0000 |
| TIB-14 | *An. malefactor* | 273 | 2 | 1 | 0.0000 | 0.0000 |
| MOÑ-186 | *An. neomaculipalpus* | 324 | 1 | 1 | 0.0000 | 0.0000 |
| MOÑ-215 | *An. neomaculipalpus* | 324 | 3 | 2 | 0.0041 | 0.0029 |
| MOÑ-220 | *An. neomaculipalpus* | 324 | 3 | 1 | 0.0000 | 0.0000 |
| MOÑ-326 | *An. neomaculipalpus* | 324 | 3 | 2 | 0.0041 | 0.0028 |
| MOÑ-337 | *An. neomaculipalpus* | 324 | 4 | 4 | 0.0046 | 0.0025 |
| TIB-43 | *An. neomaculipalpus* | 324 | 2 | 1 | 0.0000 | 0.0000 |
| TUR-51 | *An. neomaculipalpus* | 324 | 3 | 1 | 0.0000 | 0.0000 |
| ZUL-6 | *An. neomaculipalpus* | 324 | 3 | 1 | 0.0000 | 0.0000 |
| ZUL-12 | *An. neomaculipalpus* | 324 | 3 | 2 | 0.0021 | 0.0019 |
| SPU-624 | *An. neomaculipalpus* | 324 | 2 | 2 | 0.0031 | 0.0031 |
| SPU-1332 | *An. neomaculipalpus* | 324 | 1 | 1 | 0.0000 | 0.0000 |
| SPU-1341 | *An. neomaculipalpus* | 324 | 3 | 2 | 0.0041 | 0.0028 |
| NUQ-1 | *An. apicimacula* | 352 | 1 | 1 | 0.0000 | 0.0000 |
| NUQ-2 | *An. apicimacula* | 352 | 4 | 2 | 0.0030 | 0.0020 |
| NUQ-3 | *An. apicimacula* | 352 | 2 | 1 | 0.0000 | 0.0000 |
| NUQ-4 | *An. apicimacula* | 352 | 2 | 2 | 0.0060 | 0.0040 |
| NUQ-5 | *An. apicimacula* | 352 | 3 | 2 | 0.0020 | 0.0020 |
| BUE-1554 | *An. apicimacula* | 352 | 3 | 1 | 0.0000 | 0.0000 |
| NEC-01 | *An. apicimacula* | 353 | 1 | 1 | 0.0000 | 0.0000 |
| NEC-04 | *An. apicimacula* | 353 | 3 | 1 | 0.0000 | 0.0000 |
| NEC-06 | *An. apicimacula* | 353 | 3 | 3 | 0.0100 | 0.0040 |
| NEC-16 | *An. apicimacula* | 353 | 2 | 2 | 0.0170 | 0.0070 |
| TAR-138 | *An. mattogrossensis* | 336 | 2 | 2 | 0.0119 | 0.0058 |
| TAR-137 | *An. mattogrossensis* | 336 | 3 | 2 | 0.0079 | 0.0039 |
| TAR-143 | *An. peryassui* | 336 | 1 | 1 | 0.0000 | 0.0000 |
| TAR-144 | *An. peryassui* | 336 | 2 | 2 | 0.0060 | 0.0041 |
| LET-17 | *An. peryassui* | 336 | 3 | 3 | 0.0060 | 0.0033 |
