# Supplementary material for: Molecular Taxonomy Provides New Insights into Anopheles Species of the Neotropical Arribalzagia Series
Source: PLoS One. 2015 Mar 16;10(3):e0119488. doi: 10.1371/journal.pone.0119488 (PMC4361172; doi:10.1371/journal.pone.0119488)
Supplement: S2 Table — ITS2: Internal transcribed spacer 2. D: genetic distance. SE: Standard Error. K2P: Kimura 2 parameter, used as the evolutionary model. (DOCX) [file pone.0119488.s003.docx]

**Table S2. ITS2-Interspecific K2P genetic distances.**

| **Species 1** | **Species 2** | **D** | **SE** |
| --- | --- | --- | --- |
| *An. punctimacula* | *An. malefactor* | 0.115 | 0.018 |
| *An. calderoni* | *An. malefactor* | 0.142 | 0.021 |
| *An. calderoni* | *An. punctimacula* | 0.149 | 0.021 |
| *An. calderoni* | *An. neomaculipalpus* | 0.414 | 0.030 |
| *An. punctimacula* | *An. neomaculipalpus* | 0.425 | 0.030 |
| *An. neomaculipalpus* | *An. apicimacula* s.l. | 0.443 | 0.031 |
| *An. malefactor* | *An. neomaculipalpus* | 0.453 | 0.030 |
| *An. punctimacula* | *An. apicimacula* s.l. | 0.466 | 0.031 |
| *An. calderoni* | *An. apicimacula* s.l. | 0.477 | 0.031 |
| *An. malefactor* | *An. apicimacula* s.l. | 0.490 | 0.031 |
| *An. peryassui* | *An. apicimacula* s.l. | 0.552 | 0.031 |
| *An. peryassui* | *An. neomaculipalpus* | 0.574 | 0.031 |
| *An. peryassui* | *An. punctimacula* | 0.581 | 0.030 |
| *An. peryassui* | *An. calderoni* | 0.587 | 0.029 |
| *An. peryassui* | *An. malefactor* | 0.589 | 0.029 |
| *An. apicimacula* s.l. | *An. mattogrossensis* | 0.602 | 0.030 |
| *An. calderoni* | *An. mattogrossensis* | 0.619 | 0.030 |
| *An. malefactor* | *An. mattogrossensis* | 0.621 | 0.029 |
| *An. punctimacula* | *An. mattogrossensis* | 0.632 | 0.030 |
| *An. neomaculipalpus* | *An. mattogrossensis* | 0.647 | 0.029 |
| *An. peryassui* | *An. mattogrossensis* | 0.662 | 0.030 |

ITS2: Internal transcribed spacer 2. D: genetic distance. SE: Standard Error.

K2P: Kimura 2 parameter, used as the evolutionary model.
