# Supplementary material for: Molecular Taxonomy Provides New Insights into Anopheles Species of the Neotropical Arribalzagia Series
Source: PLoS One. 2015 Mar 16;10(3):e0119488. doi: 10.1371/journal.pone.0119488 (PMC4361172; doi:10.1371/journal.pone.0119488)
Supplement: S3 Table — COI: cytochrome oxidase I gene. D: genetic distance. SE: Standard Error. K2P: Kimura 2 parameter, used as the evolutionary model. (DOCX) [file pone.0119488.s004.docx]

**Table S3. *COI*-Interspecific K2P genetic distances.**

| **Species 1** | **Species 2** | **D** | **SE** |
| --- | --- | --- | --- |
| *An. punctimacula* s.s. | *An. malefactor* | 0.093 | 0.013 |
| *An. calderoni* | *An. punctimacula* s.s. | 0.097 | 0.014 |
| *An. calderoni* | *An. malefactor* | 0.100 | 0.014 |
| *An. punctimacula* s.s. | *An. apicimacula* s.l. | 0.102 | 0.012 |
| *An. punctimacula* s.s. | *An. neomaculipalpus* | 0.104 | 0.014 |
| *An. malefactor* | *An. neomaculipalpus* | 0.113 | 0.015 |
| *An. neomaculipalpus* | *An. mattogrossensis* | 0.114 | 0.015 |
| *An. malefactor* | *An. mattogrossensis* | 0.114 | 0.015 |
| *An. malefactor* | *An. apicimacula* s.l. | 0.121 | 0.014 |
| *An. punctimacula* s.s. | *An. mattogrossensis* | 0.122 | 0.016 |
| *An. calderoni* | *An. apicimacula* s.l. | 0.122 | 0.014 |
| *An. mattogrossensis* | *An. peryassui* | 0.123 | 0.016 |
| *An. punctimacula* s.s. | *An. peryassui* | 0.124 | 0.016 |
| *An. calderoni* | *An. mattogrossensis* | 0.127 | 0.016 |
| *An. malefactor* | *An. peryassui* | 0.127 | 0.016 |
| *An. neomaculipalpus* | *An. apicimacula* s.l. | 0.128 | 0.014 |
| *An. calderoni* | *An. neomaculipalpus* | 0.128 | 0.016 |
| *An. apicimacula* s.l. | *An. mattogrossensis* | 0.128 | 0.015 |
| *An. apicimacula* s.l. | *An. peryassui* | 0.137 | 0.015 |
| *An. neomaculipalpus* | *An. peryassui* | 0.140 | 0.017 |
| *An. calderoni* | *An. peryassui* | 0.147 | 0.018 |

*COI*: cytochrome oxidase I gene. D: genetic distance. SE: Standard Error.

K2P: Kimura 2 parameter, used as the evolutionary model.
